# Gridlock from diagnosis to treatment of multidrug resistant tuberculosis (MDR-TB) in Tanzania: patients’ perspectives from a focus group discussion

**DOI:** 10.1186/s12889-020-09774-3

**Published:** 2020-11-07

**Authors:** Stellah G. Mpagama, Mangi J. Ezekiel, Peter M. Mbelele, Anna M. Chongolo, Gibson S. Kibiki, Kristen Petros de Guex, Scott K. Heysell

**Affiliations:** 1 Kibong’oto Infectious Diseases Hospital –Sanya Juu Siha/Kilimanjaro Clinical Research Institute Kilimanjaro Tanzania, Mae Street, Lomakaa road, Siha Kilimanjaro, Tanzania; 2 Muhimbili University of Health and Allied Sciences, Dar es Salaam, Tanzania; 3 East African Health Research Commission, Bujumbura, Burundi; 4 Division of Infectious Diseases and International Health, University of Virginia, Charlottesville, VA USA

**Keywords:** Multidrug resistant tuberculosis (MDR-TB), Focus group discussion, Qualitative research, Molecular diagnostics, Health systems, Health care workers, Implementation

## Abstract

**Background:**

Molecular diagnostics have revolutionized the diagnosis of multidrug resistant tuberculosis (MDR-TB). Yet in Tanzania we found delay in diagnosis with more than 70% of MDR-TB patients having a history of several previous treatment courses for TB signaling prior opportunities for diagnosis. We aimed to explore patients’ viewpoints and experiences with personal and socio-behavioral obstacles from MDR-TB diagnosis to treatment in an attempt to understand these prior findings.

**Methods:**

The study was conducted in December 2016 with MDR-TB patients admitted at Kibong’oto Infectious Diseases Hospital. A qualitative approach deploying focus group discussions (FGDs) was used to gather information. Groups were sex aggregated to allow free interaction and to gauge gender specific issues in the social and behavioral contexts. The FGDs explored pathways and factors in the service delivery that may have contributed in the delay in accessing MDR-TB diagnostics and/or treatment. Collected data were coded, categorized and thematically interpreted.

**Results:**

Forty MDR-TB patients participated in six FGDs. Challenges and barriers contributing to the delay in accessing MDR-TB diagnosis to treatment were as follows: 1) Participants had a different understanding of MDR-TB that led to seeking services outside the conventional health system; 2) Socio-economic adversity made health-seeking behavior difficult and often unproductive; 3) In the health system, challenges included inadequacy of MDR-TB diagnostic centers, lack of knowledge on behalf of health care providers to consider MDR-TB and order appropriate diagnostics; 4) The specimen referral system for early diagnosis of MDR-TB was inefficient. Non-adherence of TB patients to first-line anti-TB drugs prior to MDR-TB diagnosis, given the multitude of barriers discussed, was coupled with both intentional and unintentional non-adherence of health care providers to international standards of TB care.

**Conclusion:**

Patient-centered strategies bridging communities and the health system are urgently required for optimum MDR-TB control in Tanzania.

**Supplementary Information:**

The online version contains supplementary material available at 10.1186/s12889-020-09774-3.

## Background

Multidrug resistant tuberculosis (MDR)-TB is a laboratory diagnosis that requires not only identification of *Mycobacterium tuberculosis* (MTB) but also determining resistance to at least rifampicin and isoniazid [1]. Therefore, diagnosis is either with culture and drug susceptibility testing (DST) which requires expensive biosafety or with molecular diagnostics. Consequently, processes involved in the diagnosis of MDR-TB are complicated especially in TB endemic settings where laboratory services are not universally accessible and affordable [2].

Thus, a major gap remains between the number of patients diagnosed with MDR-TB compared and the smaller number started on treatment. The World Health Organization (WHO) estimated in the year 2019, the global incidence of MDR-TB was 484,000 ranging 417,000 – 556,000. Yet in the same year, the MDR-TB global detection was 32 and 84% enrolled for treatment [3]. In TB endemic settings where health systems may be fragile, the gap is wider as illustrated in the WHO report of Tanzania for 2014. The report shows 64 persons with MDR-TB (10% of the estimated burden), of which 28 (44%) were enrolled for treatment [4]. In Tanzania several signals have suggested a considerable delay in the MDR-TB diagnostic processes to treatment, including a very high proportion of MDR-TB patients with a history of multiple episodes of TB retreatment [5–7]. Furthermore, fewer than expected people living with Human Immunodeficiency Virus (HIV) and children were diagnosed and treated for MDR-TB. The low detection and treatment gap is a primary hindrance in MDR-TB control both in minimizing morbidity and mortality but also in preventing transmission in the community**.**

Although innovations in molecular diagnostics such as XpertMTB/RIF and GenotypeMTBDRplus were considered major breakthroughs for early MDR-TB diagnosis and treatment, their impact may have been diminished as other implementation barriers prevented people with TB from realizing the potential benefit [8]. We showed in Tanzania as others have elsewhere that roll-out of molecular diagnostics had little difference in treatment outcomes compared with conventional methods despite a faster time to MDR-TB treatment initiation [7]. In response, we designed a nationwide study to investigate the challenges and bottlenecks associated with delay in diagnosis to treatment of MDR-TB, focusing on patients and the health system, in a project supported by the World Health Organization. We studied the health system through a mixed qualitative/quantitative methodology within 28 randomly selected health districts from the five highest TB burden regions in the country in 2016 and found only 40% of patients with international consensus indications for MDR-TB diagnostics had testing performed, and even fewer (30%) had results communicated back to providers or MDR-TB treatment facilities [9]. We report here the patients’ experience from MDR-TB diagnosis to treatment within that same time period. We specifically selected this qualitative research design to complement the quantitative investigations of HIV and TB clinics and diagnostic laboratories to better unravel the complexity of a problem that is dependent upon individual and community interaction yet currently addressed with rigid nationwide and regional policies during access of MDR-TB diagnosis to treatment.

## Methods

### Study design

This qualitative study used a grounded theory approach to explore patients’ perceptions and experiences throughout the various phases of MDR TB illness starting from prior to diagnosis through treatment initiation.

### Setting

Study participants were recruited from the in-patient ward at Kibong’oto Infectious Diseases Hospital (KIDH), the national centre of excellence for MDR-TB treatment in Tanzania and the first hospital to roll-out MDR-TB care in 2009. Focus groups were held in a quiet hall located within hospital premises away from the patients’ wards. The hall had adequate ventilation (to minimize transmission) while retaining privacy.

### Study participants

The study doctor reviewed the MDR-TB patients’ charts and identified the clinical characteristics of potential participants admitted in the ward using inclusion and exclusion criteria; thereafter research nurse approached the participants for discussion of the study. Participants receiving MDR-TB treatment were selected purposively and formed a focus group. Each focus group discussion (FGD) session consisted of 6–10 participants, with the aim of at least 4 separate groups for content saturation. Groups were sex segregated to allow free interaction and to gauge gender specific issues in the social and behavioral contexts. On average, most of the sessions lasted from 1 to 2 hours.

A written informed consent for FGD was provided to participants’ face- to-face. Participants were invited to attend the FGDs if meeting the following inclusion criteria: Age of 18 years or more, Karnofsky score of 60 *(defined as patient requires occasional assistance but is able to care for most of his/her personal needs)* or more, treated MDR-TB for at least 2 months, and sputum smear negative (per hospital protocol of regular sputum smear and culture to gauge treatment response). If participants could not read and write, the information sheets and consent forms were read out to them in presence of an impartial witness and a thumbprint was taken. While all FGDs were conducted at the conclusion of 2016 at KIDH; since that time Tanzania has scaled up MDR-TB services and new centers have started in 13 different regions. During the conduct of FGDs however, MDR-TB referrals were from throughout the entire country and the average bed occupancy was 60–80 per day. Despite scale up, MDR-TB treatment protocols were similar across sites [10].

### Data collection

After initial introduction only research staff remained with participants. EM and SM facilitated FGD. EM is a sociologist with postgraduate training in qualitative research methods and has extensive experience in conducting FGD in health services delivery settings in Tanzania while SM is a medical doctor with expertise in MDR-TB and clinical research. We took a systematic iterative approach to the development of FGD guide, which consisted around 5 open-ended questions (Annex I). The discussion explored patients’ experiences encountered prior to diagnosis of MDR-TB with standardized probes to query factors patients’ perceived as having contributed to diagnostic delay. We also explored TB service delivery factors that patients perceived had a negative impact in MDR-TB diagnosis and/or initiation of treatment. We sought opinion from patients on suggestions for improvement. We audio recorded the discussions with participants’ permission and a dedicated note-taker took pertinent issues for follow up as they emerged during the discussion. The digital-recorded discussions complemented with field notes and integrated decisions were transcribed verbatim in Kiswahili and thereafter translated into English (source document attached).

### Data analysis

Transcription and audio recordings were reviewed for accuracy prior to further content analysis (by EM and SM) using the conventional approach of Hsieh & Shannon [11]. Clear patterns and discrepancies that emerged were discussed and consensus was reached among the study team, in many cases with review of the audio recordings. The main themes were presented to the study participants and further consensus was reached. Themes were derived from an inductive approach and investigator’s prior theoretical understanding of MDR-TB. Data analysis was done using Nvivo Software version 8.This manuscript is reported according to the consolidated criteria for reporting qualitative research (COREQ) [12].

### Ethics

Scientific and ethical approval was obtained from the World Health Organization Ethical Review Committee and the Kilimanjaro Christian Medical College Research Ethics and Review Committee. For confidentiality audio-recorded tape and notebooks were kept confidential and only authorized people accessed the materials. The tapes were destroyed after transcription into hard copies.

## Results

Sixty-two MDR-TB patients were admitted during the period of enrollment and were available for FGDs during the same time period. Forty-five (73%) consented to participate and of those 5 did not participate due to medical severity. From the 40 total participants, 6 FGDs were arranged; 4 male and 2 female sessions were held in October 2016. All participants in groups stayed for the whole session and no negative complication was reported after the FDG. General clinical descriptors and demographics of the participants are summarized in Table 1. Major findings that contributed to a delay from MDR-TB diagnosis to treatment include inadequate knowledge of health care workers, lack of availability of rapid molecular technologies and inadequate specimen referral mechanisms. Table 2 summarizes the enablers and barriers of MDR-TB diagnosis. Findings are summarized and organized into 2 components: *patients/community* factors and *health system* factors. Incidental findings related to risk factors for developing MDR-TB are also summarized in the same manner.

**Table 1:** Distribution of demographic and clinical characteristics of patients with MDR-TB that participated in the focus group discussion

	Sub-categories	Number (%)
HIV Status	Positive	16 (40)
	Negative	24 (60)
History of previous TB treatment	None	11 (28)
	Treated TB once	13 (32)
	Treated TB twice	10 (25)
	Treated TB three times	4 (10)
	Treated TB four times	2 (5)
Region of Domicile	Dar es Salaam	11 (27)
	Geita	6 (15)
	Mbeya	5 (13)
	Others category A	8 (20)
	Others category B	10 (25)

Category A: Mwanza, Mara, Njombe and Mtwara Regions each contributed 2 participants

Category B: Morogoro, Kilimanjaro, Manyara, Shinyanga, Arusha, Singida, Sumbawanga, Tanga, Simiyu and Kigoma Regions each contributed 1 participant

**Table 2:** Summary of barriers and enablers for MDR TB diagnosis

Enablers	Barriers
Social support	Most private facilities do not test for TB
Isolation of MDR TB patients as advised by nurses	Failure to identify TB symptoms
Adherence with first-line treatment	Bread winner and single parenting- especially for female patients
Support from medical community that patient friend supported fare	Traditional healers unaware of MDR-TB
Transportation support to TB centers	Treatment without diagnostic testing
Referral for further testing of people considered for MDR-TB	Medical staff asking for money
Medical staff have mechanisms for follow-up of their patients	TB clinics located close to HIV/AIDS offices may enhance stigma
Nurse empowerment to acquire skills to be able to presume MDR-TB	Non-adherence to TB & MDR-TB treatment guidelines
Provision of TB treatment at low cost	Health care workers lack of knowledge to presume MDR-TB
Ability of patients to sacrifice finances or time for diagnosis and/or treatment	Lack of patient-centered mechanisms for specimen transport
Courage and persistence from patients to seek TB health care	

### Factors in delay from MDR-TB diagnosis to treatment

#### Patients/community factors

Examples of identified challenges and barriers contributing to delay from MDR-TB diagnosis to treatment initiation includes stigma related to HIV, lack of awareness or misperception of traditional healers regarding MDR-TB, and private facilities unable to provide diagnostic services for MDR-TB. Details summarized in Table 2 are further categorized into the following sub-themes:

##### Knowledge attitude and practice

Patients perceive that their community has limited understanding of TB in general and specifically of the additional gravity of MDR-TB. Consequently patients do not easily accept the diagnosis of MDR-TB and sought traditional healers. Others sought services at pharmaceutical shops as they provide medicines that relieve some symptoms but consequently did not receive adequate screening.

*• “I have used medications four times. Since I was still coughing, I decided to go to test at Musoma and results showed I had Chronic TB. I stayed for 1 week then I received a call that the bus has arrived and it took me to Moshi. I escaped after staying for 2 months to my mother who is staying at Simiyu. I was tested and diagnosed with Chronic TB. Thus, they brought me back to Moshi for treatment.*

*• “Honestly I don’t know regarding delaying as one may start coughing then ending up taking different medication, suspecting malaria. Until you start getting fever that’s when you go for testing and diagnosed with Chronic TB”*

*• “After it reached a point that my condition became worse my relatives decided to take me back home. At home they took me to traditional healer but the moment I ingested the given medication I vomited it as it was”.*

*• “We didn’t know I had TB, but I moved to another village thinking it was witchcraft. Since I was still coughing even after moving to another village for traditional medicine, then I opted to go to the hospital and tested sputum but I kept on believing it was witchcraft”*

*• “To blind the community who may directly link TB with HIV, majority of TB patients walk stealthily for anti-TB refill as you know TB clinics are very close to HIV clinics.”*

##### Social – economic adversity

Because of prolonged illness, financial hardships affected the majority of participants and made their health seeking difficult and often fruitless. Being unable to work for personal income during periods of diagnostic delay or unsuccessful treatment episodes had a negative impact. Several could not afford to pay for tests that ultimately resulted in delay in MDR-TB diagnosis and/or referral for treatment.

*• “They told me to go get the treatment at a nearby health center, I really had to pay for the X-ray which costed me 16000/=TSHs (~ 7.3 USD)”.*

*• “I completed 6 months of TB treatment though symptoms were persisting and I was referred to a regional hospital. I didn’t have money to cover for transport. I didn’t take my referral instead I went home to seek for support. After several days a friend of mine supported 10000 shillings (~ 4.6USD) which supported me transport and I was diagnosed as MDR-TB.”.*

*• “I am independent, I am paying rent myself, and I have a small kid and am working with Indians. This (having MDR TB) means not going for work, no job …” .*

*• “I am the day worker and I have twins younger than 7 years; I am the only one providing food and shelter, now I have MDR-TB I can’t support my family and I hesitated to start treatment”.*

#### Health system

Although there were some examples of how the health system steered to early diagnosis and referral, for example in tracing of contacts of persons previously diagnosed with TB (see quotation immediately below), the majority of participants reported struggles with the system.

*• “While I was escorting my brother, then a nurse advised us to test for TB … I was also tested positive for MDR-TB thus I also started the medication immediately”.*

**Fig. 1:**
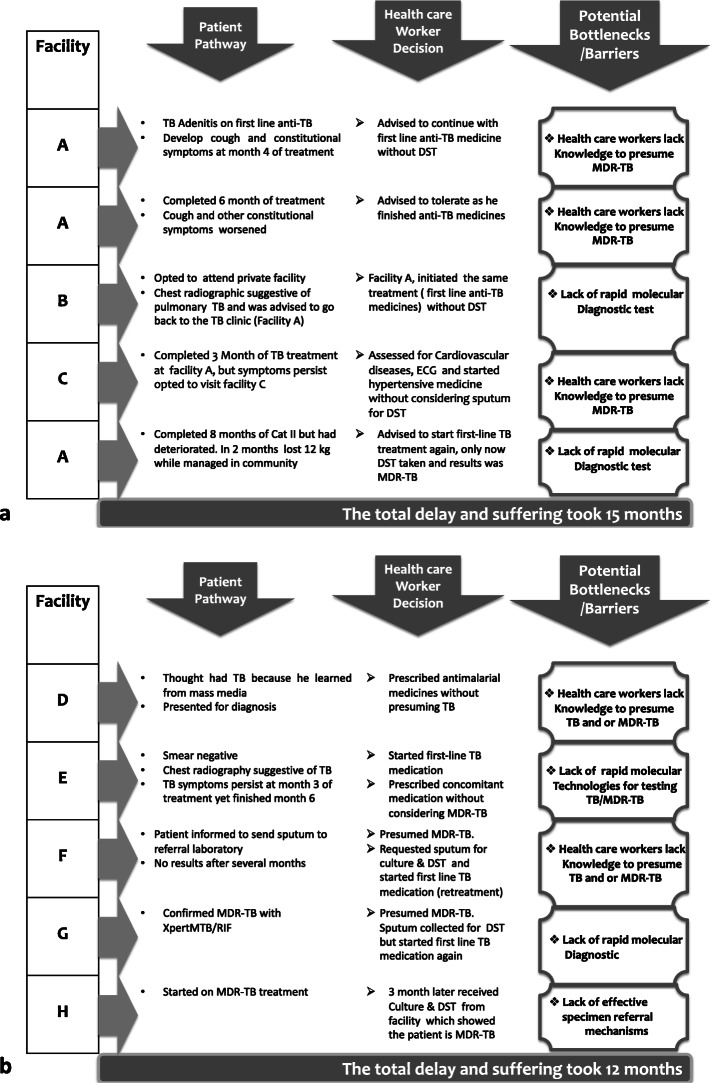
**a**: Patient has visited 3 facilities A, B and C several times encountering more than one barrier that contributed in delay in the diagnosis of MDR-TB. In between patient experienced chest pain that was mis-attributed to cardiovascular disease increasing both cost to the patient and delay in diagnosis. **b**. Patient was aware of TB symptoms yet was treated without considering a TB test. Despite visiting 4 different health facilities, several barriers prevented the diagnosis of MDR-TB. Although at Facility F MDR-TB was correctly considered and sputum collected for MDR-TB diagnosis, results were not available in a timely manner and the patient opted to shift to Facility G where diagnosis of MDR-TB was ultimately made, but the patient still required referral for eventual treatment. A full 3 month later while on MDR-TB treatment, Facility F traced the patient and communicated the MDR-TB results. DST; drug susceptibility testing

Major findings that contributed to the delay are further categorized into three sub-themes spanning from knowledge of health care providers, availability of diagnostics, and specimen referral mechanisms (Fig. 1a and b).

##### Lack of knowledge of MDR-TB among health care providers

Patients on both first-line initial treatment and retreatment regimens presented often at the hospital complaining of persistence of TB symptoms despite prolonged exposure to first-line anti-TB drugs. For example, one patient suffered prolonged first-line TB treatment and advanced from minimal nodular findings on chest x-ray to more extensive cavitary lung disease before being considered for MDR-TB testing (Fig. 1a). In some cases, providers deferred diagnostic evaluation of patient with ongoing TB symptoms despite first-line anti-TB treatment and instead suggested other diagnostic possibilities, a form of anchoring bias (Fig. 1b).

• *“I started getting fever, headache and I had severe chest pain too, I was tested and told I have Chronic TB then I was kept on treatment for six months without getting relief. Then I decide to go back to the hospital and I was given 60 injection. When I came back I was asked to collect sputum and they took the specimen to Mwanza”.*

• *“At the district the system is not good especially in testing of sputum. As one may go there for testing then they will start giving excuses that they don’t test sputum or they look at the general condition of a patient, if you don’t look sick, they will say that you are absolutely fine”.*

• *“I confronted her again; she then said for now we have to wait for the doctor to come take you to Mbeya so that you may get tested again”.*

• *“I told them I tested and results showed I have TB and I have come to take medicines. Then they told me they couldn’t put me on medication basing on the result of the place I was coming from, so I have to do another test then if the results will show I have TB then they will put me on medication. I agreed”.*

##### Inadequacy of MDR-TB diagnostic centers and inefficiency in the specimen referral system

For most patients when MDR-TB was clinically considered, they were referred to distant facilities (Fig. 1a and b). Unfortunately, patients were required to deliver their own sputum to distant laboratories, or take specimen results from a diagnostic to a treatment facility.

• *“I used to get recurrent fever, but at the private hospital where I was going they were testing me for malaria and Urinary Tract Infection. As I started coughing and getting chest tightness the doctor advised me to go at the Regional hospital for sputum test … I was diagnosed with Chronic TB and I had to go to Kibong’oto for treatment”.*

• *“They told me that I have recurrent TB and I am supposed to produce sputum for DST and send myself to the diagnostic laboratory around 100 km. However, I shouldn’t bother to follow the results because the laboratory will communicate the results to them. I travelled to that laboratory and left sputum after 3 days I was got a phone call from the hospital that I have MDR-TB”.*

• *“I was told to produce sputum for MDR-TB diagnosis while HCP were filling the laboratory form. I came back with specimen and I was told to take the specimen and the form. HCP directed me to travel to city about 50 km to send the specimen and the form. I went outside to look for friends who could support me some money for fare. I took specimen and form and travelled to city … … the following week I needed to travel the same distance to trace for results. I took the laboratory results and brought to the facility and the HCP interpreted the results that I had MDR-TB”.*

### Incidental findings that revealed the risk factors for developing MDR-TB

During discussion other patients revealed issues that were pertinent factors in both patients and the health system that may amplify MDR-TB epidemic particularly in individuals receiving first-line TB treatment.

#### Patients/community

Shared behaviors in the village such as communal locations for alcohol consumption were postulated to contribute to TB spread, but also preventable death.

• *“Honestly speaking, back in the village people are so ignorant since you might find in the local bars people share the same alcohol containers. We blow and sip from the same containers … At the end of the day we keep on spreading the disease”.*

• *“So when you find those old chronic drunkard men who drink the traditional alcohol out there in streets, it’s really hard to stop and prevent them from spreading the disease. There are so many people dying out there in the streets no joke I’m telling you”.*

#### Health system

Non-adherence to first-line drug-susceptible TB regimen was common and often due to behaviors for which the health system has little support, such as for individuals with mental health disorders and substance use. Furthermore, TB medicines are supposed to be available from the dispensaries level to the higher levels of the health system, yet facilities frequently faced shortages.

• *“I was drinking alcohol therefore there were times I stopped taking my medication so I went on like that for a while, there were weeks I was taking the medication and there were weeks I was not taking them at all”.*

• *“Sometimes you forget to take them when you get family problems while you are at home hence you might stay a week without taking medication. Or otherwise you only remember to take them when you think of the nurse who gave you the medication or perhaps when you start coughing”.*

• *“I just finished the dose without completing the injections as I was supposed to buy a syringe and I hadn’t monies. My condition got worse thus they took me to the Regional hospital where I was tested positive for MDR-TB”.*

• *“I was told if these medications finish, I should go and refill at a nearby health center within my village. After going there I was told that those particular medications are not available there” .*

• *“Where I used to go and refill my medication, sometimes they tell me they are not opening on that particular day or perhaps they say today is Sunday so we don’t give out medicines while they are the one they gave me that date”.*

• *“I had to go back to the nurse asking for her help. Then she said she is only helping me because I am sick, therefore she signed me up as a member of that region as a favor. … She filled the form and gave me the medicine and told me come back after two weeks to refill. Therefore I had been receiving the treatment for 6 months …” .*

• *“So when I reached the hospital I never found the doctor, I went there twice but unsuccessful. Unfortunately I couldn’t go anymore because of the expenses. Then I had to go back to the nurse asking for her help” .*

• *“They say even the doctors are not available or they may even say they are out off pills so they refer you … you find them closed. Unfortunately I ended up going back home without any medication. I was then admitted for Malaria treatment then I was lucky enough to be tested and confirmed positive for MDR-TB”.*

• *“At the district hospital I was asked to give sputum for testing. I stayed for one week without been given results. They were saying doctors were not around, other days I found too many patients or already closed”.*

• *“You may find him already drunk and he is saying that you should add more money at least 5000, then he gives you the medications and tells you to come back for refill and he insists that you should only go to him for the refill”.*

• *“Whenever you go back for the refill you find someone else and they start asking about your information all over again. They say they can’t see your file meanwhile your condition keeps on worsening as for some days they tell you they are out of pills.*

##### Patients’ suggestions and recommendations for improvement

Participants highlighted areas for improvement and specific mechanisms to decrease delay in MDR-TB diagnosis and treatment. Examples include an increase in education and screening in the community and improvement in availability of diagnostic technologies closer to where they seek healthcare.

• *“In the village people are diagnosed very late so if possible they should pass house by house for testing”.*

• *“Apart from using billboards, education should be provided at areas with large population or mass e.g. at the market. Most of us get TB and other transmitted diseases there”.*

• *“I think investigation should be done at the district and regional hospitals”.*

## Discussion

This study demonstrates how MDR-TB patients have been struggling tenaciously with their plight not only from the disease but also with the rigid yet porous health system. In this detailed qualitative study patients themselves have identified noteworthy challenges that are rarely discussed by providers and policy makers but which severely limit any roll-out of new technologies or expansion of treatment programs.

Participants clearly raised concerns that the majority were now being treated for MDR-TB not because of the health system, but rather in spite of it. Frontline health care workers (HCWs) routinely failed to suspect and test for MDR-TB suggesting that educational initiatives directed at HCWs may have considerable programmatic benefit. Yet diagnostic delay was common even in centers that provided TB services, contrary to other countries where patients who attended TB centers had an advantage of being suspected and screened early [13]. Likely more important is that the health system lacks efficient mechanisms for onsite specimen testing or referral of specimens and relaying of results, a common phenomenon occuring in many TB endemic countries [14], but one in which if ameliorated would concurrently raise awareness and testing for MDR-TB among the HCWs. Lack of efficient mechanisms for specimen refferals was shown to contribute to substantial delay in MDR-TB diagnosis to treatment, and this is similar to what has been reported from other settings within and outside of sub-Saharan Africa [15, 16].

Importantly, participants described corruption as a major barrier and their accounts were vividly detailed including HCWs asking for informal payment from patients for anti-TB refill and TB diagnostic tests. Some HCWs indirectly gained financial benefit from sending patients for additional tests, such as x-rays at their affiliated diagnostics centers with five fold cost increase and without providing a receipt. The consequences included non adherence and a delay in MDR-TB diagnosis and treatment as most of patients in the FGD suffered from severe economic hardship even prior to MDR-TB treatment. Corruption among those tasked for caring of patients with TB or other global health priority diseases compounds an already devastating diagnosis for an individual and on a larger scale has been likened to a biosecurity threat at the population level [17]. Our findings suggest transforming the health system into a proactive state is urgently required not only for tackling TB but also other infectious threats [18]. This will include community based diagnostic and treatment support, networking of health facilities at all levels through open access to diagnostics with transparent pricing, and strengthening public-private partnerships to shed light on individual practices. A step toward this transformation may be to apply electronic continous medical education around priority issues such as TB and tie the medical education to licensing and recertification [19].

Contextualizing both health system barriers and patient/community level factors goes far to explain why most MDR-TB treated in Tanzania still occurs for patients that have had multiple courses of first-line treatment for TB. Our findings suggest that patients survive long and complicated courses from diagnosis to treatment and may represent a form of “long-term survivor” that likely only typifies a fraction of the actual burden of MDR-TB in the community where many MDR-TB patients may have died before they access diagnosis or treatment [5].

The majority of patients did not possess a biomedical awareness of TB during their early struggles with MDR-TB diagnosis which they perceived was shared by their family and community, and rather relied on alternative belief systems of illness. Yet consistently, financial difficulty was a major barrier despite the initial belief system and included travel cost and living expenses during illness. Financial barriers were particualarly challenging for primary income earners or primary caretakers in a family. Additional identified factors that contributed to diagnostic and treatment delay were stigma and dissatisfaction with the health system. In total participants describe a state of health seeking behavior for TB that could be considerably improved but has changed little since the last national survey for such practices [20].

Patients’ perpectives of front-line HCWs suggested substantial deficiencies in providers and clinics ability to implement essential basics for preventing acquired MDR-TB and transmission [21]. One of the basics agreed upon with international consensus requires TB treatment to be standard, high quality and delivered with patient support [22]. Yet some patients confessed that they were non-adherent to the first-line anti-TB drugs and practiced anti-TB drugs holiday for alcohol substitution without knowledge of their healthcare providers that did not have capacity or ability to provide adherence support. Others reported that they did not take TB medication throughout a recommended course simply because health facilities did not have adequate TB medications in stock, which reveals another deficiency in drug supply and management which can be challenging given current centralization strategies [22]. Our patients’ experiences expose just how difficult a supervised treatment strategy can be in TB endemic countries like Tanzania that are subject to unfunded or underfunded requirements for directly oberserved therapy, which at times were passed on to the patient in the form for extra cost or general hardship. In many instances our patients’ experiences were in direct conflict with the WHO guidance on the ethics of TB prevention, care and control which advocates for patient’s autonomy, diginity and integrity [23]. Indeed, the patients’ experiences highlighted the need to rethink the necessity of directly observed therapy as it is currently recommended and focus instead on community based solutions to treatment [24]. Such community based and patients centered solutions are fitting with the current End TB Strategy which calls for engagement of all relevant stakeholders, increased access to diagnostics, treatment and care for everyone with TB as a matter of not only public health but also social justice.

The study had limitations given that FGDs were among those patients that had successfully made it to diagnosis and treatment, therefore the responses may have been limited by survival bias. The ratio of FGD men and women was 2:1 suggesting there may have been other factors driving a differential in MDR-TB diagnosis and treatment for women. One asumption might be men were prioritized by the family for treatment or diagnosed more at their place of work, for example limited TB screening efforts were underway in the mining industry. Nevertheless, the FGD did include participants that were representative of the entire country as at that time the facility was the only MDR-TB center for Tanzania. Furthermore the number of participants sampled of the total MDR-TB treatment population was relatively large. In addition, we were able to identify some factors and practices that validated our previous observations.

## Conclusion

A representative sample of people with MDR-TB shared several contextual challenges and bottlenecks that represented both patient/community and health system factors along the pathway of MDR-TB diagnosis to treatment that contributed to substantial delay with both personal and public health impact. Current roll-out of technologies like molecular diagnostics alone will not suffice in improving control of the MDR-TB epidemic. Urgent interventions directed at the patient-identified barriers could bring a greater return on TB care investment.

## Supplementary Information

**Additional file 1.**

## Data Availability

The datasets used and/or analysed during the current study available from the corresponding author on reasonable request.

## Abbreviations

FGD: Focus Group Discussion
HIV: Human Immunodeficiency Virus
MDR: Multidrug resistant
TB: Tuberculosis
WHO: World Health Organization
