# Supplementary material for: Gridlock from diagnosis to treatment of multidrug resistant tuberculosis (MDR-TB) in Tanzania: patients’ perspectives from a focus group discussion
Source: BMC Public Health. 2020 Nov 7;20:1667. doi: 10.1186/s12889-020-09774-3 (PMC7648291; doi:10.1186/s12889-020-09774-3)
Supplement: Supplementary file 1 — Additional file 1. [file 12889_2020_9774_MOESM1_ESM.pdf]

FOCUS GROUP GUIDE FOR MDR-TB PARTICIPANTS

General questions

1. What happened to you before you were diagnosed as MDR-TB?

2. What were the processes you encountered during the MDR-TB diagnosis?

3. What are things that contribute to delay in diagnosis on your side?

4. What are things that contribute to delay in diagnosis on the side of the health care settings (providers and facilities)

5. What do you recommend to make patients get early diagnosis and initiation of treatment?
